# Axicabtagene Ciloleucel versus Tisagenlecleucel for Relapsed or Refractory Large B Cell Lymphoma: A Systematic Review and Meta-Analysis

**DOI:** 10.1016/j.jtct.2024.01.074

**Published:** 2024-01-26

**Authors:** Nico Gagelmann, Michael Bishop, Francis Ayuk, Wolfgang Bethge, Bertram Glass, Anna Sureda, Marcelo C. Pasquini, Nicolaus Kröger

**Affiliations:** 1 Department of Stem Cell Transplantation, University Medical Center Hamburg-Eppendorf, Hamburg, Germany; 2 The David and Etta Jonas Center for Cellular Therapy, University of Chicago, Chicago, Illinois; 3 Department of Hematology and Oncology, University Hospital Tuebingen, Tuebingen, Germany; 4 Department of Hematology and Cell Therapy, Helios Klinikum Berlin-Buch, Berlin, Germany; 5 Bellvitge Institute for Biomedical Research, Universitat de Barcelona, Hematology Department, Institut Català d’Oncologia-Hospitalet, Barcelona, Spain; 6 Department of Medicine, Center for International Blood and Marrow Transplant Research, Medical College of Wisconsin, Milwaukee, Wisconsin

**Keywords:** DLBCL, Axicabtagene, Tisagenlecleucel, CAR-T, CRS, ICANS

## Abstract

Axicabtagene ciloleucel (axi-cel) and tisagenlecleucel (tisa-cel) are CD19-directed chimeric antigen receptor T cell (CAR-T) therapies approved for relapsed/refractory aggressive large B cell lymphoma (LBCL). Significant costs and complex manufacturing underscore the importance of evidence-based counseling regarding the outcomes of these treatments. With the aim of examining the efficacy and safety of axi-cel versus tisa-cel in patients with relapsed/refractory aggressive LBCL, we performed a systematic literature search of comparative studies evaluating outcomes in relapsed/refractory aggressive LBCL after treatment with axi-cel or tisa-cel. We calculated odds ratios (ORs) and 95% confidence intervals (CIs) for response, progression-free survival (PFS), overall survival (OS), cytokine release syndrome (CRS), immune effector cell-associated neurotoxicity syndrome (ICANS), and hematotoxicity. Meta-analysis and meta-regression were used to generate summary statistics. A total of 2372 participants were included in the 8 studies in our analysis. The dropout rate between apheresis and infusion was 13% for axi-cel versus 18% for tisa-cel, and the median time from apheresis to infusion was 32 days versus 45 days. Axi-cel showed higher odds for a complete response (OR, 1.65; *P* < .001) and was associated with higher odds for PFS at 1 year after infusion (OR, .60; *P* < .001). OS appeared to be improved with axi-cel (OR, .84; 95% CI, .68 to 1.02; *P* = .08), whereas the cumulative incidence of nonrelapse mortality (NRM) was 11.5% for axi-cel versus 3.7% for tisa-cel (*P* = .002). The main predictors for survival were lactate dehydrogenase level, Eastern Cooperative Oncology Group Performance Status, and response to bridging, and axi-cel maintained superior efficacy even in elderly patients. In terms of safety, axi-cel was associated with significantly higher odds of any-grade CRS (OR, 3.23; *P* < .001), but not of grade ≥3 CRS (*P* = .92). Axi-cel was associated with significantly higher odds of severe ICANS grade ≥3 (OR, 4.03; *P* < .001). In terms of hematotoxicity, axi-cel was significantly associated with higher odds of severe neutropenia at 1 month after infusion (OR, 2.06; *P* = .003). As a result, axi-cel was associated with significantly greater resource utilization, including prolonged hospital stay, more frequent intensive care admission, and use of agents such as tocilizumab for toxicity management. We provide strong evidence of the greater efficacy of axi-cel versus tisa-cel in relapsed/refractory aggressive LBCL. The higher toxicity and NRM seen with axi-cel might not counterbalance the overall results, highlighting the need for timely intervention and careful selection of patients, balancing resource utilization and clinical benefit.

## INTRODUCTION

Chimeric antigen receptor T cell (CAR-T) therapies targeting CD19 have shown impressive efficacy and manageable toxicity for the treatment of various lymphoma histology subtypes in the refractory setting and are now even considered in earlier treatment lines [1–4]. Tisagenlecleucel (tisa-cel) and axicabtagene ciloleucel (axi-cel) are 2 CAR-T products that were initially approved for the treatment of DLBCL in the third or subsequent line of treatment [5,6]. Both products target the same antigen but have different costimulatory domains, 4–1BB for tisa-cel and CD28 for axi-cel [2,7]. Their approval was granted after publication of the results of the pivotal JULIET and ZUMA-1 studies [5,6], and the recent update of ZUMA-1 after 5 years suggested that »40% of relapsed/ refractory patients with ≥3 lines of therapy may be cured with CAR-T [8].

These pivotal prospective results have been confirmed in retrospective data of mainly DLBCL patients from various countries. Looking at crude outcome profiles from published prospective studies suggests higher efficacy and toxicity associated with the use of axi-cel versus tisa-cel [9]; however, these observations might be prone to bias. For instance, patients with primary mediastinal B cell lymphoma were enrolled only in ZUMA-1, lymphodepletion dosing differed, and, most importantly, bridging chemotherapy for control of disease progression was allowed only in JULIET. The latter might preclude any direct comparison between the studies, as more aggressive lymphomas might urgently need bridging therapy after leukapheresis before CAR-T can be provided [10].

To account for this issue, several matching-adjusted indirect comparisons have been done to evaluate the comparative efficacy and safety of different CAR-T products [11–13]. One report suggested that axi-cel was associated with better efficacy but also with higher toxicity [11]. However, these studies have several significant problems and biases that limit their actual translation and facilitation of clinical decision making [14–16].

Therefore, in light of several recent comparative real-world studies evaluating potential differences between axi-cel and tisa-cel, in the present study we aimed to synthesize the existing evidence on the actual outcomes of axi-cel and tisa-cel in patients with relapsed or refractory DLBCL.

## METHODS

The study methodology was in accordance with recommendations from the *Cochrane Handbook* [17] and followed the PRISMA guidelines [18] and the Meta-Analysis of Observational Studies in Epidemiology (MOOSE) checklist [19].

### Study Search and Selection

We used MEDLINE and the Cochrane Central Register of Controlled Trials, in addition to other sources (www.clinicaltrials.gov, article citations). Only fully published articles were used for our quantitative analysis, to minimize reporting bias. Search terms aimed at identifying all comparative studies of axi-cel versus tisa-cel and matched-adjusted comparisons of trial data were excluded. The search was performed on May 1, 2023.

Two authors (N.G. and N.K.) independently screened the titles and abstracts of articles and then reviewed, if available, the full-length version of potentially relevant articles for eligibility. Disagreements were resolved by consulting a third author (M.B.) and then presented for joint review and consensus. Studies were eligible if they satisfied all the following characteristics: involved adult patients (age ≥18 years) receiving either axi-cel or tisa-cel; evaluated the safety and effectiveness of the CAR-T product; and reported ≥1 outcomes such as progression-free survival (PFS), overall survival (OS), relapse/progression, overall and complete response, adverse events, cytokine release syndrome (CRS), and immune effector cell-associated neurotoxicity syndrome (ICANS).

Predefined criteria for study characteristic reporting were study identification, number of patients, median age, type of disease, bridging therapy, lactate dehydrogenase (LDH) levels, Eastern Cooperative Oncology Group Performance Status (ECOG PS), and time from apheresis to actual cell infusion.

### Outcome Measures and Risk of Bias

The primary study endpoint was PFS, defined as relapse/progression or death from any cause. Secondary endpoints were OS (death from any cause), response rates (assessed within 3 months after CAR-T infusion), incidence and severity of CRS and ICANS, resource utilization in the event of toxicities, and outcomes of hematotoxicity. The severity of adverse events was graded according to the National Cancer Institute Common Terminology Criteria for Adverse Events, version 4.03, relying on reporting in each included study. CRS and ICANS were defined according to the ASTCT Consensus Grading for Cytokine Release Syndrome and Neurologic Toxicity Associated with Immune Effector Cells, if applicable [20].

We assessed possible bias using the ROBINS-I tool (Risk of Bias in Nonrandomized Studies of Interventions) tool [21]. Areas of potential bias include confounding, selection of participants, classification of intervention, deviations from intended interventions, missing data, measurement of outcomes, and selection of the reported results. Overall judgment of the risk of bias was categorized as low, moderate, serious, or critical, and the overall body of evidence was assessed using the GRADE approach (www.gradepro.org).

### Statistical Analyses

Odds ratios (ORs) and 95% confidence intervals (CIs) were calculated by pooling the results from studies using the Mantel-Haenszel method and a random-effects model. d the overall heterogeneity was assessed using the *I*^*2*^ index (with *I*^*2*^ >50% indicating moderate to high heterogeneity) [19]. In addition, a mixed-effects model was used for meta-regression to investigate associations of continuous moderator variables with the estimated study effect size [22]. With 2-tailed unpaired testing, all values with *P* < .05 were considered statistically significant. Analyses were performed using R version 4.0.5 with the meta and metafor packages [23].

## RESULTS

### Study Selection and Characteristics

**Figure 1.**
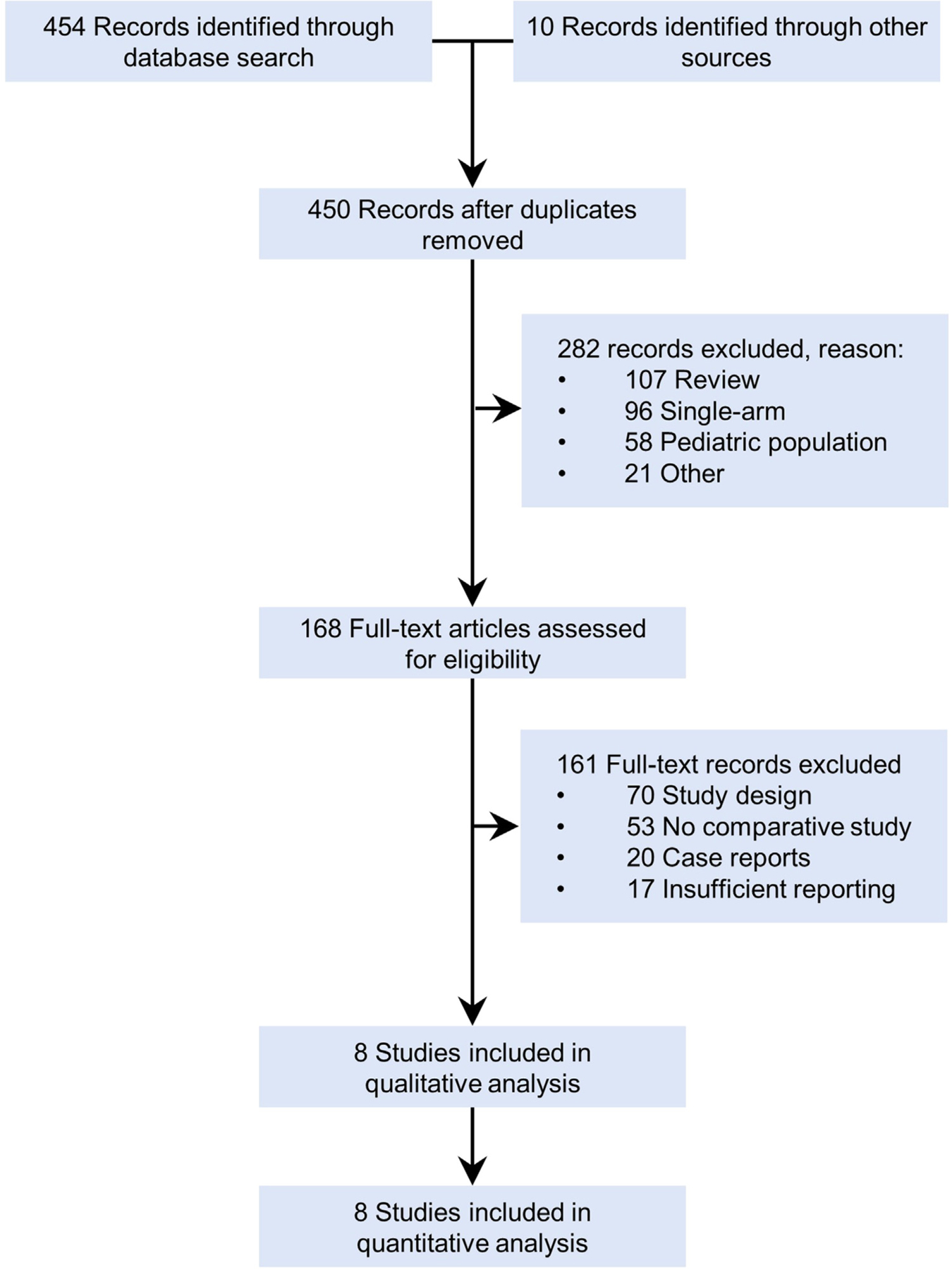
Flow chart of the study selection process.

A total of 450 unique citations were identified from the electronic database search and other sources; of these, 282 citations were excluded based on title and abstract screening and 161 were excluded based on full-text screening. Eight studies were included in our quantitative analyses (Figure 1); study characteristics are reported in Table 1. A total of 2372 participants were included in the 8 studies [24–31]. For 1 cohort, data were retrieved from 2 publications reporting results for the same population [27,32]. All studies were retrospective comparative (ie, “real-world”) studies. One study used propensity score matching to adjust comparisons [29]. Reporting of disease type and histology was heterogenous, and approximately 75% of patients in both the axi-cel and tisa-cel groups had DLBCL NOS (not otherwise specified). Four studies were evaluable for the rate of dropout between the time of apheresis and the time of actual CAR-T infusion, including a total of 1780 patients, of whom 1530 (86%) eventually received an infusion [24,25,29,30]. The dropout rate was 13% for the axi-cel group versus 18% for the tisa-cel group. The remaining studies reported data only for infused patients [26–28].

In the present analysis, 1277 patients were infused with axi-cel and 817 patients received tisa-cel. Among the infused patients, the median age was 60 years for axi-cel recipients and 64 years for tisa-cel recipients (*P* < .001). The median time from apheresis to CAR-T infusion was 32 days for the axi-cel group versus 45 days for the tisa-cel group (*P* < .001).

### Risk of Bias and Quality Assessment

Assessment revealed a low risk of bias in 4 studies [24,25,29,30], a moderate risk of bias in 2 studies [26,27], and serious risk of bias in 1 study [28]. Overall, the risk of bias of the included studies was low, as summarized in Supplementary Table S1. The main dimensions for possible risk of bias were missing data and patient selection because of the retrospective nature of the studies.

In accordance with the GRADE approach, quality assessment started at a moderate quality of evidence after downgrading, owing to the retrospective design of the included studies. The overall quality of evidence ranged from low to high (Supplementary Table S2).

### Responses

**Figure 2.**
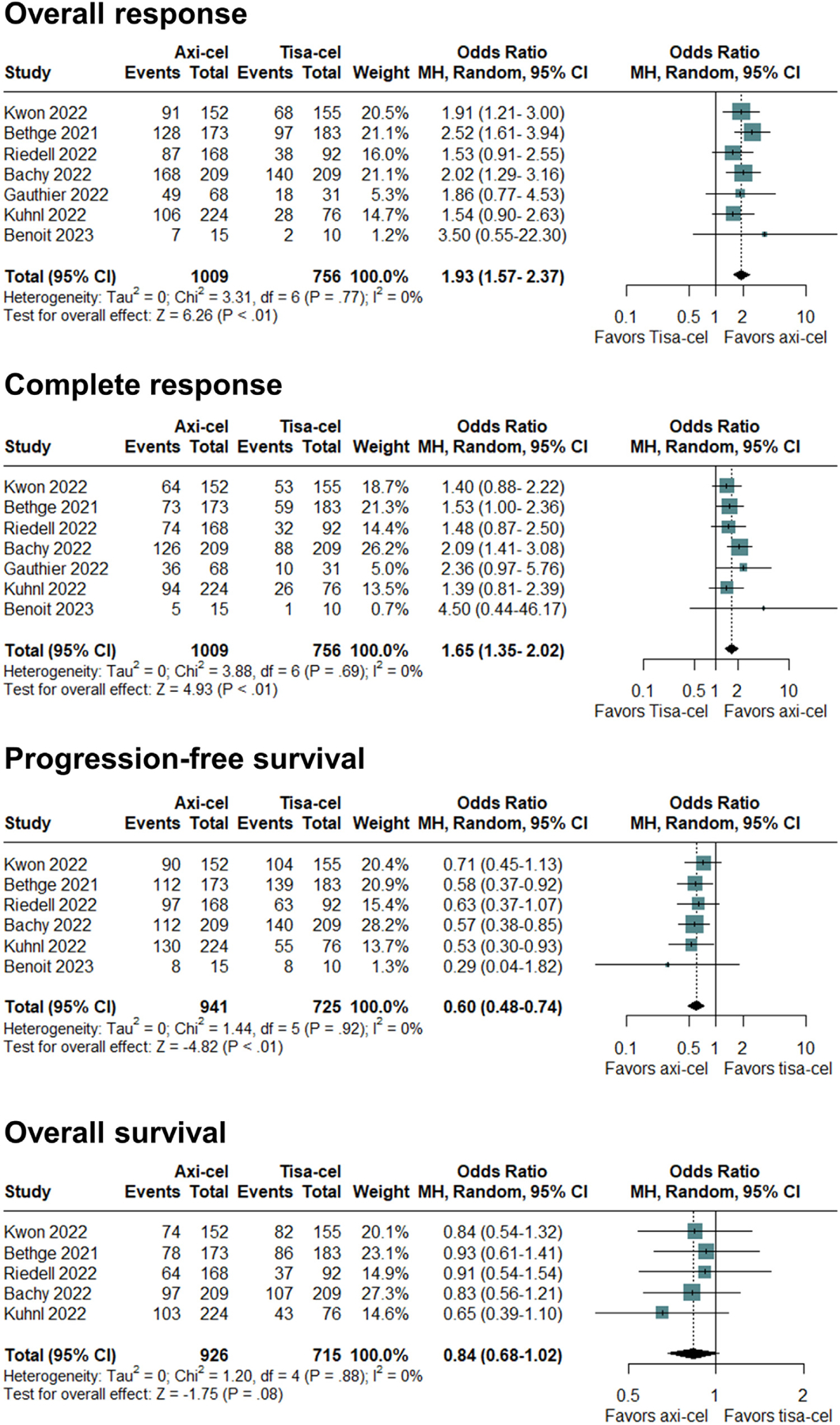
Efficacy outcomes of axi-cel vs tisa-cel in terms of overall response, complete response, PFS, and OS.

Responses within 3 months after CAR-T infusion were assessed in 7 of the 8 studies. The overall response rate differed significantly between the axi-cel and tisa-cel groups (*P* < .001), showing an OR of 1.92 (95% CI, 1.57 to 2.37) in favor of axi-cel (Figure 2A). No heterogeneity was observed (*I*^*2*^ = 0%), and the quality of evidence was moderate. There was no significant difference between the groups in the duration of response, with similar response rates at 1 year (*P* = .42). The axi-cel group also had higher odds of complete response (*P* < .001), resulting in an OR of 1.65 (95% CI, 1.35 to 2.02). No heterogeneity was observed (*I*^*2*^ = 0%), and the quality of evidence was moderate (Figure 2B).

### Survival and NRM

In terms of survival outcomes, PFS was assessed in 6 studies that included 1666 patients. Axi-cel was associated with higher odds of PFS at 1 year (*P* < .001), with an OR of .60 (95% CI, .48 to .74). No heterogeneity was observed (*I*^*2*^ = 0%), and the quality of evidence was moderate (Figure 2C). OS at 1 year was assessed in 5 studies including 1641 patients. Axi-cel appeared to be associated with higher odds of improved OS (*P* = .08), showing an OR of .84 (95% CI, .68 to 1.02). No heterogeneity was observed (*I*^*2*^ = 0%), and the quality of evidence was low (Figure 2D).

NRM was assessed in 4 studies including 1223 patients. We found a significant difference in NRM (*P* = .002), showing an OR of 2.40 (95% CI, 1.38 to 4.16) in favor of tisa-cel. No heterogeneity was observed (*I*^*2*^ = 0%), and the quality of evidence was high (Supplementary Figure S1). The cumulative incidence of NRM was 11.5% in the axi-cel group versus 3.7% in the tisa-cel group (*P* = .002).

### Predictors of Efficacy

A qualitative review was done for predictors of survival in view of heterogeneous reporting, specifically for the comparison of CAR-T products. Four studies assessed predictors of outcomes in multivariable models, and 1 study was a propensity score- matched analysis assessing other effects on outcome apart from CAR-T product in univariate analyses. In summary, the main predictors for PFS and OS were LDH, ECOG PS, and response to bridging (Supplementary Table S3). Two studies identified axi-cel as an independent predictor of better PFS. The type of CAR-T product was not included in the multivariable model of 1 study. Two studies showed significantly improved PFS with axi-cel across age groups, even suggesting that older patients might have performed better with axi-cel than younger patients (Supplementary Table S3).

### Toxicity

**Figure 3.**
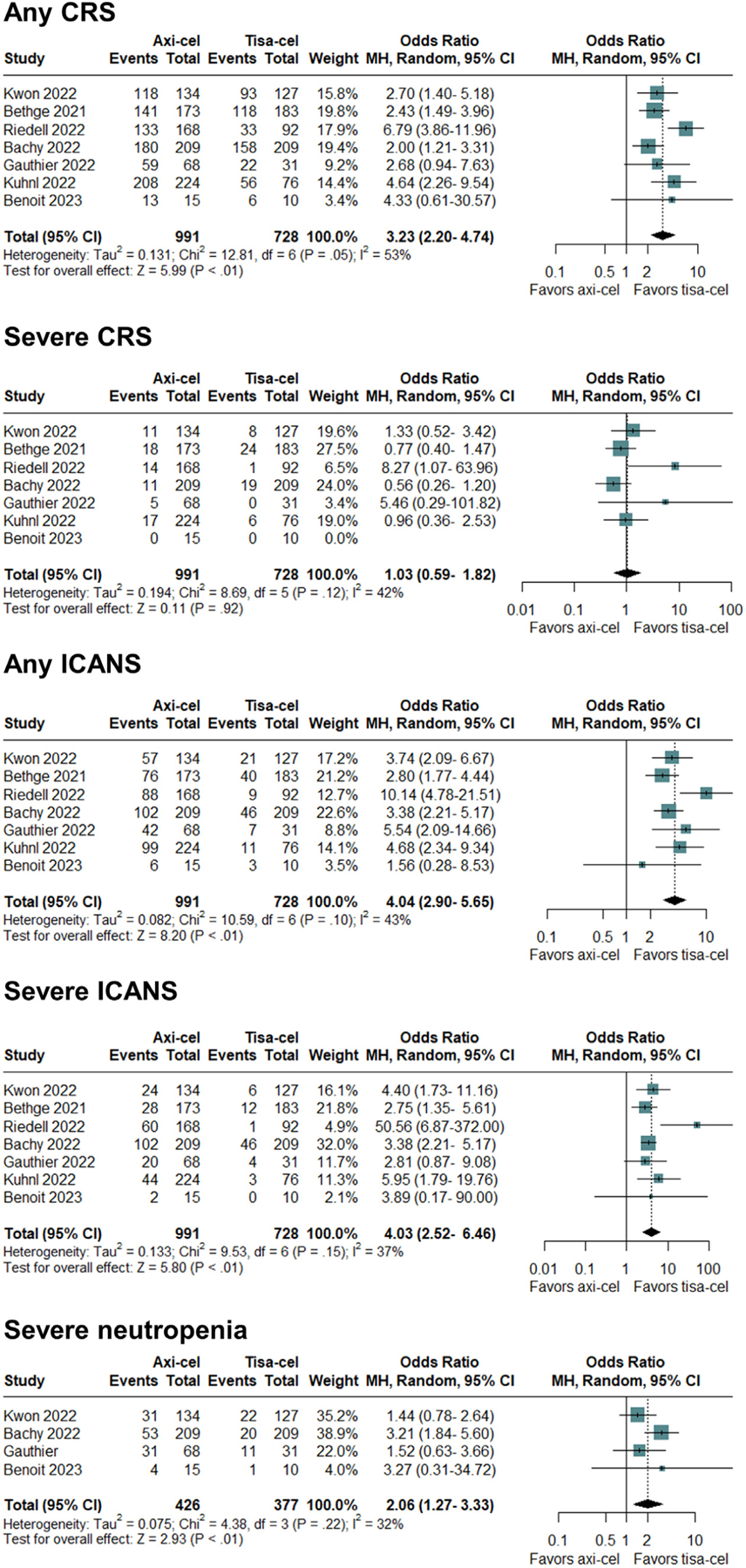
Safety outcomes of axi-cel versus tisa-cel in terms of CRS of any grade, CRS grade ≥3, ICANS of any grade, ICANS grade ≥3, and severe neutropenia at 1 month after CAR-T infusion.

Outcomes for CRS were assessed in all 8 studies. Seven studies used the ASTCT criteria, and 1 study used the Lee criteria [28]. Axi-cel was associated with significantly higher odds for occurrence of CRS of any grade (*P* < .001), with an OR of 3.23 (95% CI, 2.20 to 4.74) in favor of tisa-cel (Figure 3A). Moderate heterogeneity was observed (*I*^*2*^ = 53%; P = .05), and the quality of evidence was high. In terms of severe toxicity, no significant difference between the 2 groups was observed for CRS grade ≥3 (*P* = .92), with an OR of 1.03 (95% CI, .59 to 1.82). No significant heterogeneity was observed (*I*^*2*^ = 42%; *P* = .12), and the quality of evidence was moderate (Figure 3B). The mean duration of CRS was 5.5 ± .6 days for the axi-cel group versus 4.5 ± .6 days for the tisa-celgroup.

All studies assessed outcomes in terms of neurotoxicity. Axi-cel was significantly associated with the occurrence of ICANS of any grade (*P* < .001), with an OR of 4.04 (95% CI, 2.90 to 5.65) in favor of tisa-cel (Figure 3C). No significant heterogeneity was observed (*I*^*2*^ = 43%; *P* = .10), and the quality of evidence was high. Only 1 single-center study found no significant difference in ICANS of any grade. In terms of severity, axi-cel was associated with significantly higher odds of severe ICANS grade ≥3 (*P* < .001), showing an OR of 4.03 (95% CI, 2.52 to 6.46) in favor of tisa-cel (Figure 3D). No significant heterogeneity was observed (*I*^*2*^ = 37%; *P* = .16), and the quality of evidence was moderate. The mean duration of ICANS was 5.5 ± 1.3 days for the axi-cel group versus 4.75 ± 1.7 days for the tisa-cel group.

Hematotoxicity was assessed in 5 studies. No significant difference between the 2 groups was seen in thrombocytopenia grade 3–4 at 1 month postinfusion (*P* = .11), showing an OR of 1.68 (95% CI, 0.89 to 3.18). Moderate heterogeneity was observed (*I*^*2*^ = 62%; *P* = .05), and the quality of evidence was low. For persistent severe thrombocy-topenia at 3 months after infusion, the OR was 1.92 (95% CI, .80 to 4.65; *P* = .15). For neutropenia, axi-cel was significantly associated with higher odds of severe neutropenia grade 3–4 at 1 month after infusion (*P* = .003), showing an OR of 2.06 (95% CI, 1.27 to 3.33) in favor of tisa-cel (Figure 3E). No heterogeneity (*I*^*2*^ = 32%; *P* = .22) was observed, and the quality of evidence was high.

### Resource Utilization

We investigated potential differences in resource utilization affected by different efficacy and safety outcomes. The mean duration of hospitalization for axi-cel was 20.5 ± 2.1 days for axi-cel versus 17 ± 1.4 days for tisa-cel (*P* = .03), with respective ranges of 15 to 29 days and 10 to 22 days. The numbers of 1 study were driven by the majority of patients (64%) receiving tisa-cel infusion in the outpatient setting. Axi-cel was associated with higher odds of intensive care unit (ICU) admission (*P* = .03), showing an OR of 3.28 (95% CI, 1.16 to 9.30) in favor of tisa-cel. The median duration of ICU admission was 4 days (range, 0 to 19 days) for the axi-cel group versus 2 days (range 0 to 15 days) for the tisa-cel group. Moderate heterogeneity was observed (*I*^*2*^ = 82%; *P* = .004), and the quality of evidence was moderate. In association with safety outcomes, axi-cel was significantly associated with higher odds for tocilizumab, dexamethasone, and anakinra use (P < .001 for all) (Table 2).

## DISCUSSION

In this study of outcomes after CAR-T with axi-cel or tisa-cel for patients with relapsed or refractory LBCL, we found significantly improved response rates and PFS in patients who received axi-cel. OS also appeared to be associated with odds in favor of axi-cel, whereas NRM appeared to be higher after axi-cel treatment. In terms of CAR-T-specific toxicities, although axi-cel showed higher odds for occurrence of CRS of any grade, we found no significant difference between the 2 products for severe CRS grade ≥3. However, axi-cel was consistently associated with the occurrence of ICANS of any grade and, more importantly, of severe events. In terms of hematotoxicity, axi-cel also was associated with severe neutropenia at 1 month after infusion and thus with higher odds of greater resource utilization during the course of treatment.

We found a difference in dropout rate from the time of approval/apheresis and CAR-T infusion between the 2 products (13% for axi-cel and 18% for tisa-cel), whereas an effect of cohort origin was noted in terms of CAR-T turnaround times, being longer in European cohorts compared to US cohorts, as noted previously [33,34]. This theoretically may affect dropout rates in the intention-to-treat population and might have a negative impact on efficacy in patients who present with a more proliferative, high-burden disease at the time of infusion. The time to infusion did not impact outcomes, which might reflect anticipated delays for patients who received bridging therapy and had stable disease. Overall, there was no heterogeneity across the studies in terms of efficacy outcomes, and in general, our results demonstrate that (1) more than 1 in 10 patients who are selected and eligible for CAR-T are never infused, and (2) rapid turnaround times should be achieved for best outcomes for each individual patient.

In terms of efficacy, much of the difference in PFS between the 2 CAR-T products might have been related to the proportion of patients achieving a response to axi-cel compared to patients treated with tisa-cel. The factor most consistently associated with durable long-term remission following CAR-T is the depth of the initial response to treatment [35], and we found especially higher odds for achieving a complete response within the first 3 months after infusion with axi-cel across the studies. Furthermore, responses might be affected by CAR-T product design, expansion, and persistence, as well as by disease characteristics [9]. A 4–1BB-based autologous anti-CD19 CAR-T product like tisa-cel is known to lead to longer persistence of the CAR-T cells in vivo, but a CD28 costimulatory domain has been shown to lead to greater and faster proliferation [36]. In addition, it has been previously demonstrated that for axi-cel, peak CAR-T cell measures, normal platelet count at the start of lymphodepletion, and no prior stem cell transplantation are associated with better PFS [37]. For tisa-cel, recently reported data indicate a potential dose-response relationship between tumor burden before infusion and subsequent disease control, suggesting that tisa-cel might be more potent in DLBCL cases with a lower tumor burden [38]. However, in a subgroup analysis, no difference in efficacy between the 2 products was documented in patients with and without bulky disease at lymphodepletion [29]. Moreover, in 2 studies using LDH as a potential proxy for tumor burden and CAR-T product, multivariable analysis showed an independent risk for worse outcomes for tisa-cel. Further correlations using total metabolic tumor volume, not available for analysis in the present study, will be of interest to further dissect potential differences and thereby individualize decision making.

Regarding toxicity, axi-cel was associated with significantly more frequent low-grade CRS and, more importantly, with significantly more frequent severe neutropenia at 1 month after infusion and grade ≥3 ICANS. Of note, the possible underreporting of severe toxicities in retrospective studies included in this analysis cannot be excluded. On the other hand, it can be hypothesized that better understanding of mechanisms, prediction of risk, and new mitigation strategies for CRS and ICANS, but also hematotoxicity, will lead to much lower rates of severe events [34,39–43]. For instance, recent data from a prospective evaluation of early use of dexamethasone after axi-cel infusion demonstrated a 17% incidence of grade ≥3 ICANS [44]. Moreover, marked and prolonged hematologic toxicity can be addressed by close follow-up and timely use of a hematopoietic stem cell boost, leading to significantly improved outcomes in patients with subsequent hematopoietic recovery [39]. Therefore, even if higher efficacy with axi-cel comes at the cost of higher toxicity and slightly higher NRM, the latter might not undermine the significantly better overall outcome. Importantly, these results also were recently shown in patients receiving axi-cel as second-line treatment [45]. In summary, these results suggesting the superiority of axi-cel even in elderly patients are of immediate relevance, as real-world studies have shown a preference for tisa-cel in older and comorbid patients [46].

Because toxicity might be of greater concern in elderly patients and could counterbalance the higher efficacy, subgroup analyses of these patients are of clinical relevance. Two studies included subgroup analyses in patients age ≤70/ 65 years and those age >70/65 years [27,29,32]. Importantly, greater efficacy of axi-cel was observed consistently across age categories. Another a study evaluating patients age ≥75 years found even more pronounced differences between axi-cel and tisa-cel in these patients, with a 1-year PFS of 38% versus 15%, respectively, but no significant difference in OS was noted [32].

Our results are generally in line with another recently published comparative meta-analysis [47] but also show some differences, related mostly to differences in methodology. First, and reassuringly, both analyses generally support better efficacy in terms of overall response rate and PFS with axi-cel and a better safety profile, especially in terms of neurotoxicity, for tisa-cel. Second, as recommended by current guidelines on conducting systematic reviews and meta-analyses [19], we focused on published real-world studies that directly compared the 2 products in quantitative fashion. In contrast, Jacobson et al. [47] also included noncomparative studies and prospective trials and also applied matched-adjusted comparisons. Although this approach increases the total number of patients included in analysis, bias might be introduced by higher heterogeneity, duplication of patients, and potential reporting bias owing to different follow-up and patient characteristics. Furthermore, matched-adjusted comparisons are prone to obvious biases such as artificial controls, maximizing differences rather than aiming to account for them [14,48]. Third, Jacobson et al. used time-to-event analysis for outcomes in PFS and OS, creating hazard ratios (HRs) that enable actual risk statements for progression and/or death from any cause, finding adjusted HRs for OS and PFS of .60 and .67, respectively, both in favor of axi-cel. Confidence intervals were nearly the same for both endpoints. Such creation of adjusted HRs is mostly appropriate for prospective trials [49], as time-to-event outcomes are specifically followed up, whereas in retrospective studies (the predominant type included here), survival data are timepoint outcomes. Thus, significant assumptions are needed to enable data transformation into HRs (time-to-event data and the proportional hazards assumption), which may introduce a high risk of bias toward maximization rather than control of differences between studies and study cohorts. For instance, as we show in our qualitative analysis of univariate and multivariate analyses of studies, confounders differed significantly differed across the studies (Supplementary Table S3). In the analysis by Jacobson et al. [47], survival results might have been driven mainly by the study from Bachy et al. [29], which applied propensity score matching and provided the most comprehensive time-to-event results. Absolute OS rates in other registry studies were not statistically significant, suggesting a potential for attrition bias [50].

Fourth, our analysis included the potential differences in resource utilization that might drive cost-effectiveness [51,52] and thereby product selection in regions with limited financial capability or access [53], showing a significantly longer duration of hospitalization and higher ICU admission rates for axi-cel. Finally, our analysis represents an academic project, whereas the analysis by Jacobson et al. is an industry-led endeavor. This might have certain advantages, including access to original trial data, but it also increases the risk of reporting bias [50,54], as most recently observed with cost-effectiveness analyses [51,55,56].

In summary, both analyses provide sufficient information on better responses and PFS with axi-cel, whereas tisa-cel was associated with a better safety profile. Although OS might be improved with axi-cel for many patients, our analysis highlights the need to identify certain subgroups that might still benefit from tisa-cel in terms of both safety and efficacy, and underscores that such studies might aid clinical practice in resource-limited settings.

### Limitations

Synthesis of evidence remains essential for informing current practice, showing overall efficacy and safety results of certain treatments while highlighting the heterogeneity and caveats of current data. Our study evaluated so-called “real-world” cohorts, given the lack of sufficient prospective randomized trials comparing CAR-T products. Unfortunately, however, these studies are not likely to be conducted. Therefore, syntheses of real-world studies are important to assess whether conclusions are reproducible in routine practice and whether they can be translated to a more diverse patient population across countries and healthcare systems. Furthermore, real-world studies can provide a critical basis for comparative analyses. However, real-world studies may be prone to biases that might be transported and even worsened when synthesizing these results. We aimed to systematically assess the best possible basis for evidence generation using analysis of heterogeneity, detailed assessment of potential risk of bias, and overall quality assessment. Two studies showed a serious risk of bias [28,31], mostly related to small sample size in both studies, affecting sufficient data and outcome reporting. One study focused mainly on resource utilization and reported efficacy and safety outcomes as secondary objectives, which might have introduced attrition and selection bias [31]. Nonetheless, both reports provide a full picture of current evidence, which is an important objective of systematic review and meta-analysis to transparently inform clinicians and patients alike [19,57].

By including mainly retrospective data, this analysis relied on timepoint evaluations, whereas post-CAR-T information, such as subsequent therapies (eg, transplantation, salvage regimens), was not available in most studies. CAR-T can be used as bridging for transplantation, or transplantation can be performed as salvage therapy in relapsed patients after CAR-T. One study of transplantation post-CAR-T found a median interval of 4 months between treatments, with a high incidence of hepatic toxicity [58]. Two-year NRM was approximately 25%, and OS was 45%. Such data might suggest that transplantation after anti-CD19 CAR-T failure is feasible but with possibly high toxicity [58,59]. Furthermore, in cases with insufficient information for more subtle analysis of subgroups or potential confounders, we listed findings from each study for transparency. Overall, in terms of main endpoints, heterogeneity across studies was not observed, and the quality of evidence was moderate to high, suggesting reliable evidence. Thus, our results have clear advantages over matched-adjusted indirect comparisons of trial data with retrospective cohorts, as highlighted elsewhere [60].

## CONCLUSION

Our study provides strong evidence of the greater efficacy of axi-cel versus tisa-cel; however, the higher toxicity and NRM seen with axi-cel might not counterbalance the overall results and highlight the need for more careful screening and timely intervention for these patients. This study also highlights the need for adequate reporting of study results and may facilitate clinicians’ choice of CAR-T product for a specific patient, balancing safety and efficacy.

## Supplementary Material

1

## Figures and Tables

**Table 1:** Characteristics of the Included Studies

Study	Number of Patients		Age, yr, median		DLBCL, %		Prior Lines of Therapy, median (range)/(%)		Days from Apheresis to Infusion		Bridging, %		Prior SCT, %		LDH > Normal, %		ECOG PS 0–1, %	
	Axi-cel	Tisa-cel	Axi-cel	Tisa-cel	Axi-cel	Tisa-cel	Axi-cel	Tisa-cel	Axi-cel	Tisa-cel	Axi-cel	Tisa-cel	Axi-cel	Tisa-cel	Axi-cel	Tisa-cel	Axi-cel	Tisa-cel
Bethge et al., 2022 [27]	173	183	60	61	88	93	≥3 (67)	≥3 (74)	35	55	72	84	33	35	65	55	84	84
Bachy et al., 2022 [29]	494	315	63	64	74	78	2 (2–8)	3 (2–10)	NR		82	86	21	26	55	50	86	82
Kwon et al., 2023 [25]	152	155	59	62	75	64	2 (2–6)	2 (2–7)	NR		78	83	31	29	50	60	95	93
Gauthier et al., 2022 [26]	68	31	62	64	74	58	3 (2–4)	3 (2–4)	27	40	59	71	NR		NR		NR	
Benoit et al., 2023 [28]	15	10	59	67	67	60	≥3 (0)	≥3 (5)	28	36	44		47	40	NR		100	
Kuhnl et al., 2022 [24]	292	112	57	63	64	75	≥3 (37)	≥3 (42)	40	50	88	82	18	12	71	73	90	91
Riedell et al., 2022 [30]	168	92	59	67			3 (2–10)	4 (2–9)	28	45								
Mian et al., 2023 [31]	55	29	<65: 65/41		100	100	≥4 (42)	≥4 (34)	NR		NR		45	24	NR		75	76

NR indicates not reported.

**Table 2:** Resource Utilization for Toxicity Management

Study	Tocilizumab, %		Dexamethasone, %		Anakinra, %	
	Axi-cel	Tisa-cel	Axi-cel	Tisa-cel	Axi-cel	Tisa-cel
Kwon et al., 2023 [25]	60	31	31	9	9	2
Gauthier et al., 2022 [26]	49	42	60	16		
Benoit et al., 2023 [28]	87	30	87	20		
Kuhnl et al., 2022 [24]	74	47	44	25		
Riedell et al., 2022 [30]	61	13	54	10		
Mian et al., 2023* [31]	1 (0–5)	0 (0–2)				

* Only the median number and range of tocilizumab doses were given.

## Data Availability

The datasets used and/or analyzed in this study are available from the corresponding author on reasonable request.
