# Supplementary material for: Axicabtagene Ciloleucel versus Tisagenlecleucel for Relapsed or Refractory Large B Cell Lymphoma: A Systematic Review and Meta-Analysis
Source: Transplant Cell Ther. Author manuscript; Available in PMC 2025 Jan 27. (PMC11771143; doi:10.1016/j.jtct.2024.01.074)

Supplement for

**Axicabtagene ciloleucel versus tisagenlecleucel for relapsed or refractory large B-cell lymphoma: a systematic review and meta-analysis**

Nico Gagelmann^1^, Michael Bishop^2^, Francis Ayuk^1^, Wolfgang Bethge^3^, Bertram Glass^4^, Anna Sureda^5^, Marcelo C Pasquini^6^, Nicolaus Kröger^1^

1 Department of Stem Cell Transplantation, University Medical Center Hamburg-Eppendorf, Hamburg, Germany

2 The David and Etta Jonas Center for Cellular Therapy, University of Chicago, Chicago, Illinois, United States

3 Department of Hematology and Oncology, University Hospital Tuebingen, Tuebingen, Germany

4 Department of Hematology and Cell Therapy, Helios Klinikum Berlin-Buch, Berlin, Germany

5 Bellvitge Institute for Biomedical Research, Universitat de Barcelona, Hematology Department, Institut Català d'Oncologia-Hospitalet, Barcelona

6 Department of Medicine, Center for International Blood and Marrow Transplant Research, Medical College of Wisconsin, Milwaukee, WI

**Table 1. Risk of bias of included studies.**

| **Study** | **Confounding** | **Selection of participants** | **Classification of participants** | **Deviations from intended interventions** | **Missing data** | **Measurement of outcomes** | **Selection of reported results** | **Overall risk of bias** |
| --- | --- | --- | --- | --- | --- | --- | --- | --- |
| Bethge | Yes | No | PN | Yes | PN | Yes | Yes | Moderate |
| Bachy | Yes | Yes | Yes | Yes | PN | Yes | Yes | Low |
| Kwon | Yes | Yes | Yes | Yes | PN | Yes | Yes | Low |
| Gauthier | Yes | No | PN | Yes | PN | Yes | PN | Moderate |
| Benoit | No | No | PN | Yes | PN | Yes | PN | Serious |
| Kuhnl | Yes | Yes | Yes | Yes | PN | Yes | Yes | Low |
| Riedell | Yes | Yes | Yes | Yes | PN | Yes | Yes | Low |
| Mian | No | No | PN | Yes | PN | Yes | PN | Serious |

**Table 2. Quality assessment.**

| **No. of studies** | **Risk of bias** | **Inconsistency** | **Indirectness** | **Imprecision** | **Publication bias** | **Axi-cel** | **Tisa-cel** | **Odds ratio (95% CI)** | **Quality** | **Importance** |
| --- | --- | --- | --- | --- | --- | --- | --- | --- | --- | --- |
| **Overall response** | | | | | | | | | | |
| 7 | Low | Not Serious | Not serious | Not serious | NA | 1009 | 755 | 1.93  (1.57-2.37) | ⨁⨁⨁◯  Moderate | Critical |
| **Complete response** | | | | | | | | | | |
| 7 | Low | Not Serious | Not serious | Not serious | NA | 1009 | 755 | 1.65  (1.35-2.02) | ⨁⨁⨁◯  Moderate | Critical |
| **Progression-free survival** | | | | | | | | | | |
| 6 | Low | Not Serious | Not serious | Not serious | NA | 941 | 725 | 0.60  (0.48-0.74) | ⨁⨁⨁◯  Moderate | Critical |
| **Overall survival** | | | | | | | | | | |
| 5 | Low | Not Serious | Not serious | Serious^a^ | NA | 926 | 715 | 0.84  (0.68-1.02) | ⨁⨁◯◯  Low | Critical |
| **Non-relapse mortality** | | | | | | | | | | |
| 4 | Low | Not Serious | Not serious | Not serious | NA | 785 | 532 | 2.40  (1.38-4.16) | ⨁⨁⨁⨁  High | Critical |
| **CRS** | | | | | | | | | | |
| 7 | Low | Serious^b^ | Not serious | Not serious | NA | 991 | 728 | 3.23  (2.20-4.74) | ⨁⨁⨁⨁  High | Critical |
| **ICANS** | | | | | | | | | | |
| 7 | Low | Not Serious | Not serious | Not serious | NA | 991 | 728 | 4.04  (2.90-5.65) | ⨁⨁⨁⨁  High | Critical |
| ^a^Estimates crossing decision threshold  ^b^ I²=53%, P=0.05 | | | | | | | | | | |

**Table 3. Predictors of outcome and subgroup analysis according to CAR-T product.**

| **Study** | **Predictors for worse PFS** | **Predictors for worse OS** | **Subgroup analysis on PFS** |
| --- | --- | --- | --- |
| Bethge  (2021) | MVA:  Bridging non-responder vs responder  LDH >normal vs normal  Increasing ECOG  Tisa-cel vs axi-cel  Younger age | MVA:  Bridging non-responder vs responder  LDH >normal vs normal  Increasing ECOG | ≥65 years:  axi-cel 45% vs tisa-cel 28% (P=0.05)  <65 years:  axi-cel 30% vs tisa-cel 22% (P=0.05) |
| Bachy  (2022) | UVA:  No prior transplant vs prior transplant  DLBCL/HGBL vs tFL/MZL  Time from last treatment ≤91 vs >91 days  Bulk vs no bulk  Stage III-IV vs I-II  Center effect  ECOG ≥2 vs 0-1  Bridging non-responder  CRP >30 vs ≤30mg/L  LDH >normal vs normal | UVA:  No prior transplant vs prior transplant  DLBCL/HGBL vs tFL/MZL  Time from last treatment ≤91 vs >91 days  Bulk vs no bulk  Stage III-IV vs I-II  ECOG ≥2 vs 0-1  Bridging non-responder  CRP >30 vs ≤30mg/L  LDH >normal vs normal  Male vs female sex | >70 years:  axi-cel 64% vs tisa-cel 48% (P=0.003)  ≤70 years:  axi-cel 55% vs tisa-cel 43% (P=0.01)  Bulk ≤5cm:  axi-cel 57% vs tisa-cel 48% (P=0.02)  Bulk >5cm:  axi-cel 59% vs tisa-cel 31% (P=0.002) |
| Kwon  (2022) | MVA:  Progressive disease vs other  LDH >normal vs normal  ECOG ≥2 vs 0-1 | MVA:  Progressive disease vs other  LDH >normal vs normal  ECOG ≥2 vs 0-1 |  |
| Kuhnl  (2022) | MVA:  LDH >normal vs normal  Extranodal sites ≥3 vs <3 | MVA:  LDH >normal vs normal  Increasing ECOG  Platelets <50 vs ≥50 |  |
| Riedell  (2022) | MVA:  Tisa-cel vs axi-cel  LDH >normal vs normal  Progressive disease vs primary refractory  Peak ferritin ≥5000 ng/mL | MVA:  Tisa-cel vs axi-cel  LDH >normal vs normal  Progressive disease vs primary refractory  Peak ferritin ≥5000 ng/mL |  |

**Figure 1. Non-relapse mortality.**


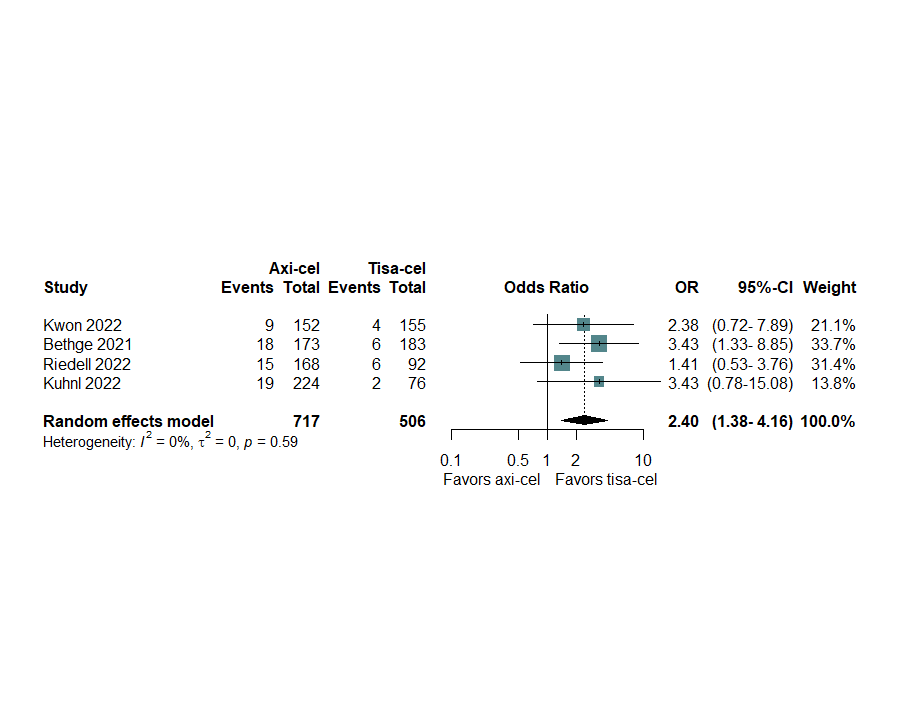

Supplement: 1 [file NIHMS2041785-supplement-1.docx]
